# Degradation of poly aromatic fractions of crude oil and detection of catabolic genes in hydrocarbon-degrading bacteria isolated from Agbabu bitumen sediments in Ondo State

**DOI:** 10.3934/microbiol.2019.4.308

**Published:** 2019-10-22

**Authors:** Temitayo O. Olowomofe, J. O. Oluyege, B.I. Aderiye, O. A. Oluwole

**Affiliations:** 1 Department of Microbiology, Ekiti State University, Ado-Ekiti; 2 Department of Science and Laboratory Technology, Ekiti State University, Ado-Ekiti

**Keywords:** bioremediation, crude oil, poly aromatic hydrocarbon, gas chromatography/mass spectroscopy, biodegradation

## Abstract

Pollution due to release of Poly aromatic hydrocarbons (PAHs) are a major environmental issue especially in oil producing communities. This study investigates the polyaromatic hydrocarbon degradation potentials of some bacteria: *Campylobacter hominis, Bacillus cereus, Dyadobacter koreensis, Pseudomonas aeruginosa* and *Micrococcus luteus* isolated from Agbabu bitumen sediments in Ondo State. The isolates were used singly and in consortium for the degradation of Bonny light crude oil. Concentrations of residual aromatic hydrocarbons in crude oil degraded by these isolates were determined by Gas chromatography/Mass Spectroscopy with flame ionization detector (FID). Detection of catabolic genes (*nahH, CatA and AlkB*) in the isolates was determined by PCR amplification of their specific primers. The GC-MS analyses showed degradation of poly aromatic hydrocarbons (PAHs) by these isolates. The consortium exhibited the highest PAH reduction (73%) while *C. hominis* had the least PAH reduction (56%). *Dyadobacter koreensis, P. aeruginosa, Micrococcus luteus and B. cereus*, displayed 66%, 60%, 59% and 58% PAH reduction respectively. The catabolic gene *nahH* gene was present in *B. cereus, D. koreensis, P. aeruginosa* and *M. luteus, alkB* gene was present in *B. cereus, C. hominis*, and *D. koreensis* while *CatA* was not detected in any of the isolates. The findings of this study affirmed the hydrocarbon-degrading abilities and presence of catabolic genes in these bacteria, these make them potential tools in oil prospecting and cleaning up of hydrocarbon contaminated sites.

## 1. Introduction

Pollution caused by crude oil and its derivatives are the most widespread problem in aquatic and terrestrial ecosystems and are a huge source of environmental concern worldwide [1]. The Niger Delta region of Nigeria known for its oil deposits has had a devastating experience of oil spills on both the terrestrial and aquatic environments in the past 50 years of crude oil exploration and production [2]. The region is among the ten most significant wetland and marine ecosystems in the world that has been severely damaged by petroleum pollution due to unsustainable oil exploration activities [3]. A major concern during petroleum hydrocarbon pollution is the presence of heavy compounds such as polycyclic aromatic hydrocarbons (PAHs), asphaltenes and many branched compounds with twenty or more carbon atoms which are relatively resistant to biodegradation and may move up marine food chains and taint fishes or shellfishes, which further can be used as food or feed, and thus can harm higher life forms through biomagnifications [4],[5]. PAHs are common petroleum contaminants in the environment considered to be potentially mutagenic and carcinogenic [6],[7].

Current conventional remediation approaches include physicochemical techniques such as photo-oxidation, burying, dispersion, washing, incineration, thermal conversion and other pyrolysis techniques [8]. However, their high cost of application and the resultant toxic intermediates being generated make them largely unsuitable. Bioremediation, defined as the use of microorganisms to detoxify or remove pollutants is regarded as a preferable alternative due to its low cost, high efficiency, environmental friendliness and simplicity technology for long term restoration of crude oil contaminated sites [9]. The technology utilizes the metabolic potential of microorganisms such as bacteria, fungi and few protozoa in degrading liquid petroleum spilled on terrestrial and marine environments, into harmless compounds [10]. Being known to be hydrocarbonoclastic [11], microorganisms offer an alternative solution to the problems of clean up [12].

The biodegradation of PAHs in soil is completed in two steps. First step involves uptake of PAHs by soil microbes, which is affected by several factors such as the bioavailability of PAHs in soil (aqueous phase), properties of soil, and environmental conditions. The second step involves degradation of PAHs by microbes, which mostly depends on the biological ability of microbes [13],[14]. Oil-eating microorganisms produce enzymes which break down hydrocarbon compounds through single or multiple metabolic pathways. The enzymes produced are incapable of breaking down all forms of hydrocarbon compounds; as a result, most oleophilic microbes are hydrocarbon-specific, although a few are physiologically-versatile and can degrade a wide-range of hydrocarbons [15]. It is uncommon to find organisms that could effectively degrade both aliphatics and aromatics possibly due to differences in metabolic routes and pathways for the degradation of the two classes of hydrocarbons. However, some reports have suggested the possibility of bacterial species with propensities for degradation of both aliphatic and aromatic hydrocarbons simultaneously [16].

In the terrestrial as well as aquatic ecosystems, the environmental fate of PAHs is determined by the degree of their degradation by microbes. Several PAHs contaminated soil and sediments have active populations of PAHs degrading microorganisms. Oboh *et al.* [1] in their investigation showed that some bacteria species isolated from bitumen deposit had crude oil degradative potentials and as such are suitable for degradation studies due to their ability to utilize hydrocarbon as sole carbon source. Since the Nigerian bitumen possesses relatively large quantity of naphthenes, aromatics and asphaltenes that are similar to the conventional oil, the isolates recovered from there should also possess PAHs degradation properties [17]. Also, commencement of commercial exploitation of the bitumen is likely to introduce large quantities of both PAHs and PCBs into the environment through anthropogenic activities.

Since no sufficient information has been reported on the ability of indigenous bacteria in Agbabu bitumen deposit to degrade poly aromatic hydrocarbons and also the mechanism whereby microorganisms degrade hydrocarbons has not been fully elucidated, this study therefore aimed at evaluating the PAHs degradative capabilities and presence of catabolic genes in five bacteria strains indigenous to Agbabu bitumen sediments. Knowledge of the degradative potentials of these bacteria will thus provide baseline data which are essential in remediation of these toxic pollutants in the environment.

## 2. Materials and methods

### 2.1. Source of isolates

The five isolates used in this study were previously characterized via 16S rRNA sequencing as *Campylobacter hominis, Bacillus cereus, Dyadobacter koreensis, Pseudomonas aeruginosa* and *Micrococcus luteus* were recovered from bitumen-contaminated sites in Agbabu, Ondo State and have been reported to be hydrocarbonoclastic [18],[19].

### 2.2. PAH degradation studies

The PAH degrading potentials of these five isolates singly and in consortium was evaluated in this study. The consortium used was Consortium (*Bacillus cereus*, *Dyadobacter koreensis, Pseudomonas aeruginosa, Campylobacter hominis* and *Micrococcus luteus)*. The isolates stored on slant were reactivated overnight in nutrient broth, the harvested cultures were reduced to microbial load of 0.7 OD_600nm_ which was inoculated into mineral salt medium (0.2 g of KCl, 6 g of Na_2_HPO_4_, 2.8 g of NaH_2_PO_4_, 0.1 g of MgS0_4_ and 5 g of NaCl) containing 2% (v/v) crude-oil. The set up was incubated at 30 °C for 21 days. The optical density (OD_600nm_) was measured at seven days interval using spectrophotometer (JENWAY 6705).

### 2.3. Analysis of residual crude oil after degradation

The residual crude oil in the culture fluids after degradation was extracted at seven days interval using the method of Orhorhoro *et al.* [20]. The extracted oil was fractionated by liquid-solid chromatography using a column packed with activated silica gel G-60 to separate the aromatic and the aliphatic components [21]. The aromatic fractions were analyzed by Hewlett Packard 5890 Series II GC /MS equipped with flame ionization detector (FID) and 30 m long HP-5 column (internal diameter, 0.25 mm; film thickness, 0.25 µm) method described by Olabemiwo *et al*. [17].

Percentage reduction=(Concentration of Control−residual concentration experiment/Concentration of control)×100

### 2.4. PCR amplification of catabolic genes from the Isolates

The presence of catabolic genes: catechol 2,3 dioxygenase (*nahH*), alkane monooxygenase (*alkB*) and catechol 1,2 dioxygenase *(CatA*) genes in the PAH-degrading bacteria species was determined via PCR amplification using the primers 2,3D_zewF (ATGAAAAAAGGCGTAATGCGC) and 2,3D_zewR (AGCACGGTCATGAAACGTTCGTTC for *nahH* while primers AlkBF(5′CCTGCTCCCGATCCTCGA3′) AlkBR (5′TCGTACCGCCCCGCTGTCCAG3′) and C12O F 5′ (GCCGCCACCGACAAGTT-3′) and C12O R (5′-CACCATGAGGTGCAGGTG-3′ were used to amplify alkB and CatA genes respectively [22],[23].

### 2.5. Statistical analysis

The data generated from this study were analyzed using Microsoft excel and Chi- square of the Statistical Procedure for Social Science version 22.0 (SPSS, Chicago, IL, USA).

## 3. Results

### 3.1. Concentration of PAH in residual degraded crude oil from Pseudomonas aeruginosa culture medium

**Figure 1.**
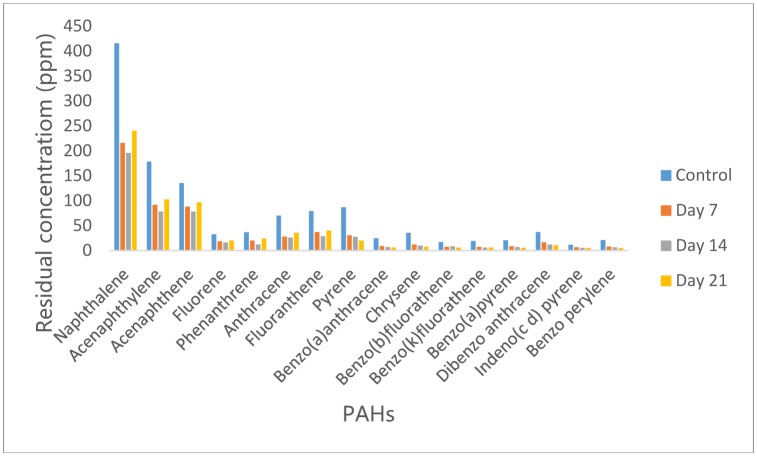
Concentrations of PAH in residual crude-oil from *Pseudomonas aeruginosa* culture medium.

Figure 1 shows the residual concentrations of Poly aromatic hydrocarbons (PAHs) in *P. aeruginosa* inoculated crude oil supplemented MSM after 21 days incubation period in comparison with the control sample. Sixteen PAHs grouped as 2 to 6 ring PAHs respectively were found in the control following GC analyses. In the *P. aeruginosa* degraded oil, it was observed that 2-3 ring PAHs viz Naphthalene, Acenaphthene and Acenaphthylene had relatively higher concentrations than the 4–6 ring PAHs (Fluorene, Phenanthrene, Anthracene, Fluoranthene, Pyrene, Benzo(a)anthracene, Chrysene, Benzo(b)fluorathene, Benzo(k)fluorathene, Benzo(a)pyrene, Indeno(1,2,3-cd) pyrene, Dibenzo (a,h)anthracene and Benzo(g,h,i) perylene). However, treatment with *Pseudomonas aeruginosa* strain led to reduction in the concentrations of all the PAHs after 7 days incubation with Chrysene the most degraded with 65% reduction while Acenaphthene was the least degraded compound with 35% reduction in concentration. Further reduction in the PAHs concentrations was observed at day 14 with Chrysene, had reduced by 72% while Acenaphthene had reduced 1.2 times further than day 7. As the incubation period increased to 21 days, there was increase in the concentration of 2–4 ring PAHs while 5 and 6 ring PAHs reduced in concentration. *P. aeruginosa* had achieved 57% reduction in total PAHs concentration after 14 days of incubation. Analysis of variance showed that no significant difference existed in the concentrations of PAHs on day 7, 14, 21 and the control.

### 3.2. Concentration of PAH in residual crude oil from Bacillus cereus culture medium

**Figure 2.**
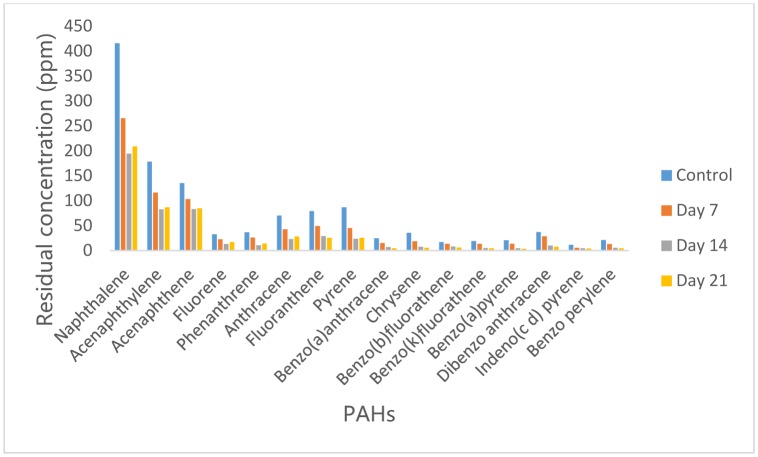
Concentrations of PAH in residual crude oil from *Bacillus cereus* culture medium.

Analysis by GC/MS revealed that *Bacillus cereus* was capable of degrading most of the Poly-aromatic hydrocarbon compounds in the crude oil used in this study. The concentrations of PAHs in residual crude oil from *Bacillus cereus* culture medium are shown in Figure 2. An insignificant reduction (p > 0.05) in the concentrations of PAHs was observed on day 7, di benzo (a,h) anthracene had the highest percentage reduction (50%) while Benzo (b) fluoranthene had the lowest percentage reduction of 21%. Further significant reduction (p < 0.05) in the PAHs concentrations was observed after 14 days where highest percentage reduction of 79% was recorded in benzo (a) pyrene. Other PAHs with relatively high reduction were Phenanthrene, Pyrene, Benzo (a) anthracene, Chrysene, Benzo (k) fluoranthene, indeno (1,2,3, c d) pyrene and Benzo (g,h,i) perylene with 71%, 73%, 72%, 78%, 73%,73% and 75% reduction respectively while Acenaphthylene had the lowest reduction of 39%. Further reduction in the concentrations of PAHs was observed after 21 days except for naphthalene acenaphthylene, acenaphthene, fluorene, phenanthrene, anthracene and pyrene whose concentrations increased from 194.6 ppm, 82.89 ppm, 82.99 ppm, 13.12 ppm, 10.73 ppm, 23.12 ppm and 23.58 ppm to 209.07 ppm, 86.53 ppm, 84.82ppm, 16.85 ppm, 14.08 ppm, 28.13 ppm and 25.50 ppm respectively.

### 3.3. Concentration of PAH in residual crude oil from Dyadobacter koreensis culture medium

**Figure 3.**
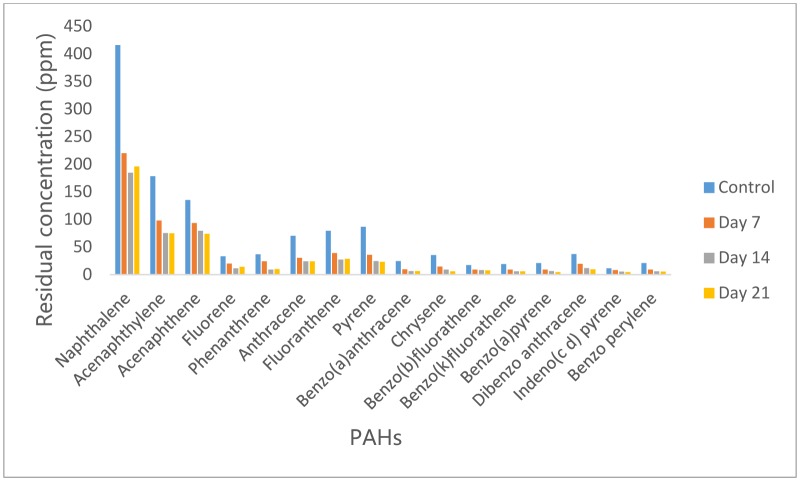
Concentrations of PAH in residual crude oil from *Dyadobacter koreensis* culture medium.

The data presented in figure 3 showed that inoculation of Bonny Light crude oil with *Dyadobacter koreensis* led to reduction in the concentrations of all the PAHs on day 7 as compared with that of control. Statistical analysis however revealed that no significant difference exist between the concentrations of PAHs in control and on day 7. However, as the incubation period increased to 14 days, significant reduction (p < 0.05) in their concentration was observed. Chrysene had highest percentage reduction of 83%, Phenanthrene, Pyrene, Benzo(a) anthracene, Benzo (a) pyrene and di benzo (a,h) anthracene had relatively high percentage reduction of 75%, 73%, 74%, 78%, 74% and 75% respectively while Acenaphthene had the least reduction of 41%. After 21 days, further reduction though insignificant (p > 0.05) compared to that obtained on day 14 was observed for some PAHs, majorly the 5 and 6 ring PAHs while concentrations of 2 and 3 ring PAHs increased (Figure 3).

### 3.4. Concentration of PAH in residual crude oil from Campylobacter hominis culture medium

**Figure 4.**
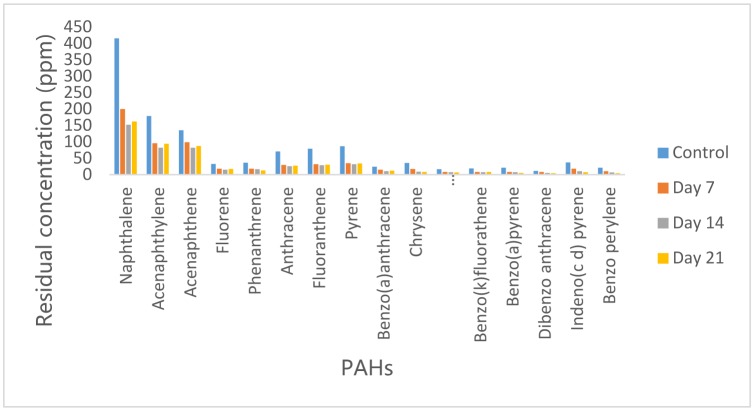
Concentrations of PAH in residual crude oil from *Campylobacter hominis* culture medium.

Application of *Campylobacter hominis* resulted in the reduction of PAHs concentration on day 7 with 69% of the PAHs analyzed having about 50% of their initial concentration reduced (Figure 4). Statistical analysis revealed that there is no significant difference in the PAHs concentrations in control and after 7 days. On the 14^th^ day of incubation, further reduction in their concentrations was observed. Indeno (1,2,3 c d) pyrene had the highest percentage reduction of 72% while Acenaphthene had the lowest reduction of 39%. After 21 days, further reduction in the concentrations of 5 and 6 ring PAHs was observed while concentrations of 2,3 and 4 ring PAHs increased.

### 3.5. Concentration of PAH in residual crude oil from Micrococcus luteus culture medium

**Figure 5.**
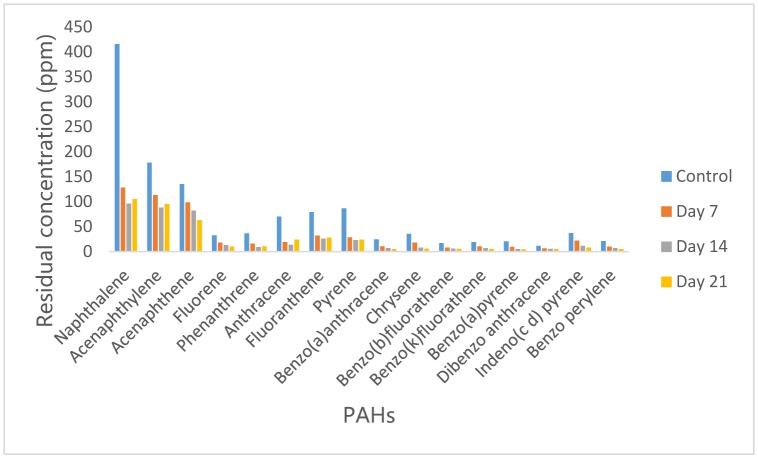
Concentrations of PAH in residual crude oil from *Micrococcus luteus* culture medium.

Inoculation of *Micrococcus luteus* did not lead to complete removal of any of the PAHs but appreciable reduction in their concentrations was observed. Eight out of the sixteen PAHs detected in the control sample had over 50% reduction in their concentrations on the 7^th^ day of incubation. Analysis of variance showed that there was significant reduction (p < 0.05) in their concentrations after 14 days where 81% of the total PAHs analyzed had over 60% of their concentrations reduced. However, highest percentage reduction of 80% was observed in Anthracene while Acenaphthene had the lowest reduction of 30%. Concentrations of 5 and 6 ring PAHs reduced further after 21 days while concentrations of 2,3 and 4 ring PAHs increased albeit insignificantly (Figure 5).

### 3.6. Concentration of PAH in residual crude oil from Consortium (Bacillus cereus, Dyadobacter koreensis and Pseudomonas aeruginosa, Campylobacter hominis and Micrococcus luteus) culture medium

**Figure 6.**
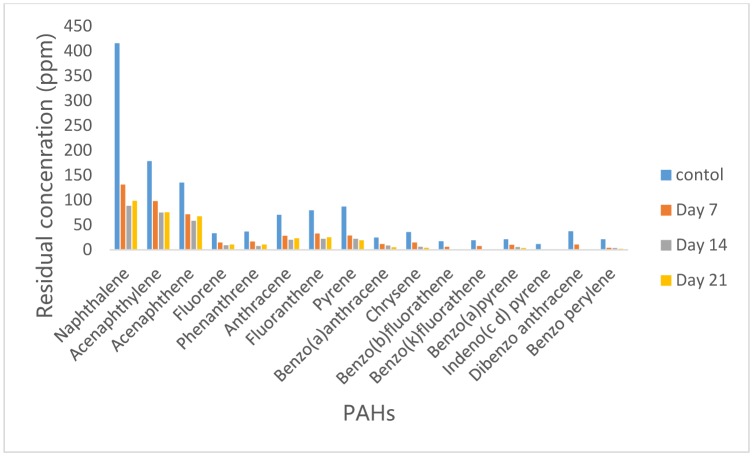
Concentrations of PAH in residual crude oil from Consortium (*Bacillus cereus*, *Dyadobacter koreensis and Pseudomonas aeruginosa, Campylobacter hominis* and *Micrococcus luteus*) culture medium.

Inoculation of the five hydrocarbon-degrading bacteria combined resulted in the total removal of Indeno (1,2,3, c d) pyrene, Benzo (k) fluoranthene, Benzo (b) fluoranthene and dibenzo (a,h) anthracene and significant reduction in the concentrations of other PAHs. However, on the 7^th^ day, all the PAHs lost more than half of their original concentrations except dibenzo (a,h) anthracene which was totally removed. As the incubation period increased to 14 days, Indeno (1,2,3, c d) pyrene, Benzo (k) fluoranthene and Benzo (b) fluoranthene had been completely removed while concentrations of other PAHs reduced significantly (p < 0.05). After 21 days, the remaining large PAHs (5–6 ring) had further reduction in their concentrations while the 2 to 4 ring PAHs concentrations increased indicating a breakdown of the larger compounds (Figure 6).

**Figure 7.**
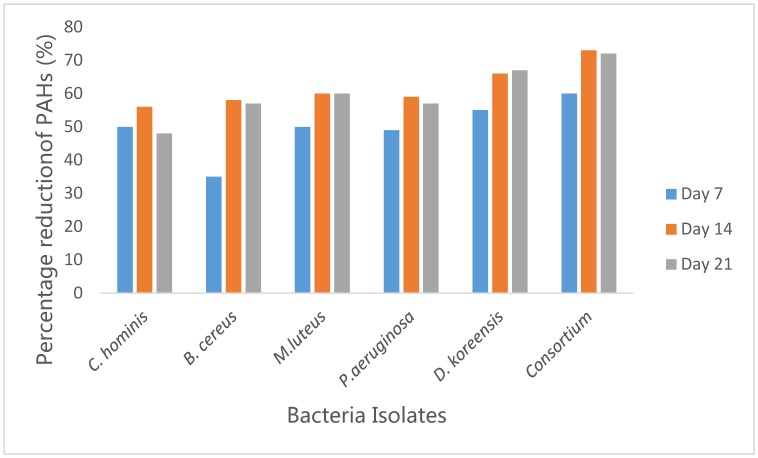
Percentage reduction of PAHs by the five hydrocarbon-degrading bacteria and their Consortium.

### 3.7. Detection of catabolic genes in hydrocarbon-degrading bacteria.

**Figure 8.**
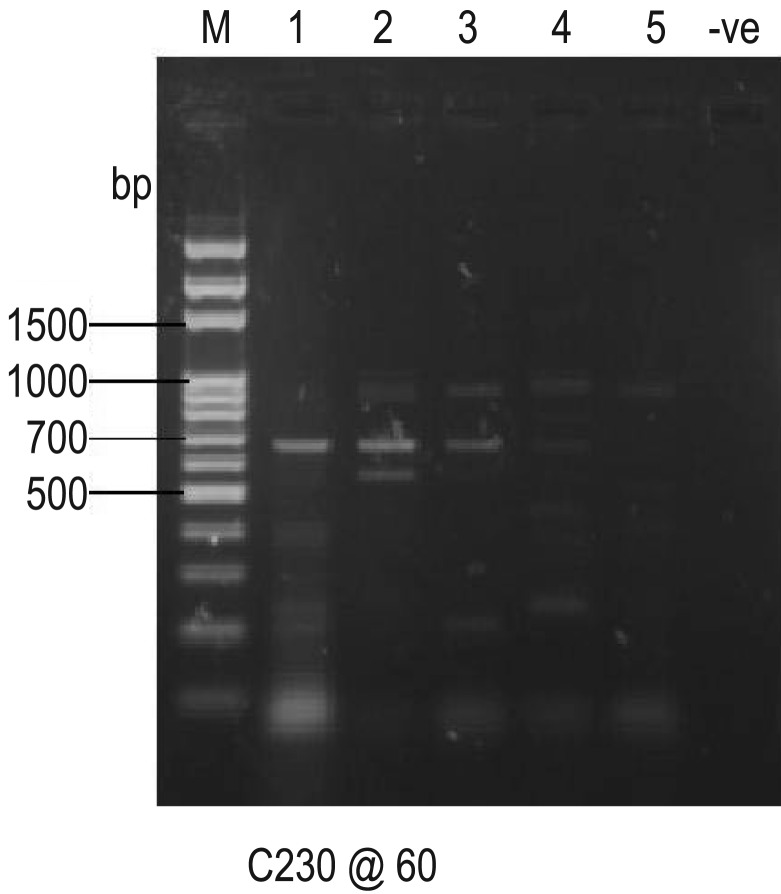
PCR Amplification of catechol 2,3 dioxygenase (*nahH*) gene primer by selected hydrocarbon-degrading bacteria. Key: M: Molecular marker; -ve: Negative control; 1: *Campylobacter hominis*; 2: *Bacillus cereus*; 3: *Dyadobacter koreensis*; 4: *Pseudomonas aeruginosa*; 5: *Micrococcus luteus*

**Figure 9.**
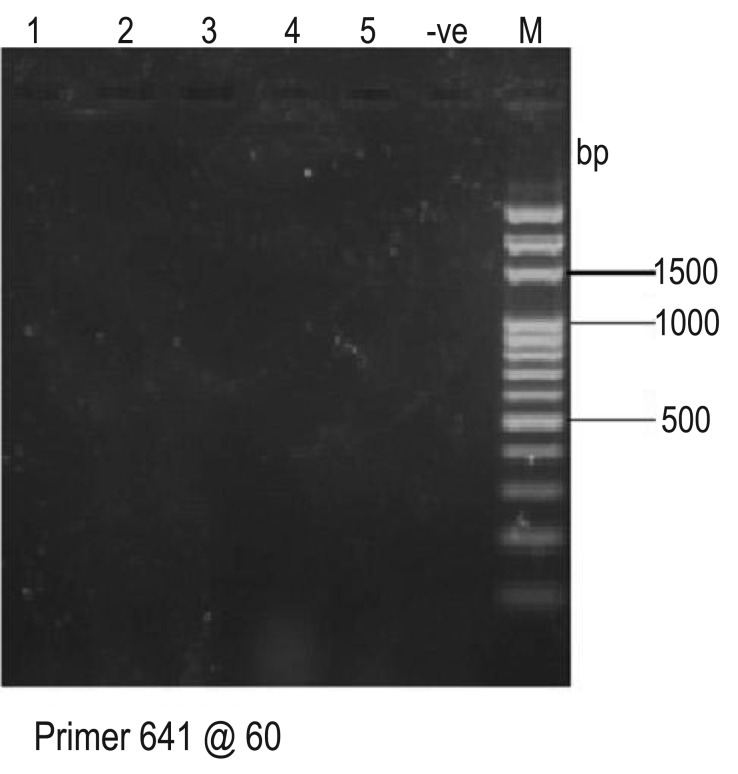
PCR Amplification of *CatA* gene primer by selected hydrocarbon-degrading bacteria. Key: M: Molecular marker; -ve: Negative control; 1: *Campylobacter hominis*; 2: *Bacillus cereus*; 3: *Dyadobacter koreensis*; 4: *Pseudomonas aeruginosa*; 5: *Micrococcus luteus*

**Figure 10.**
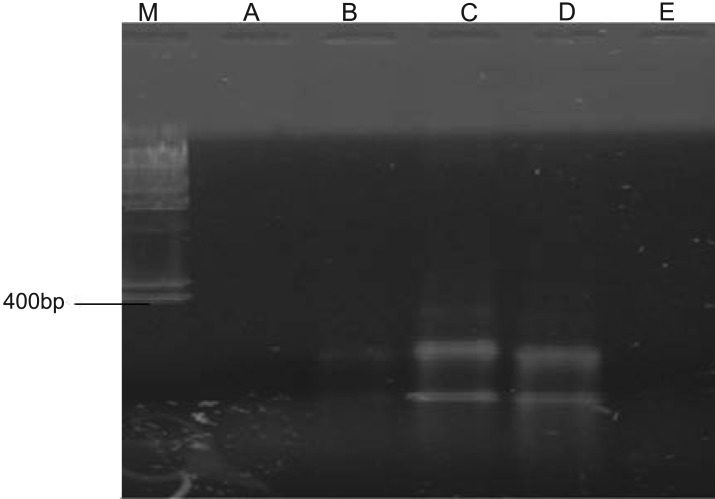
PCR Amplification of *AlkB* gene primer by selected hydrocarbon-degrading bacteria. Key: M: Molecular marker; -ve: Negative control; A: *Campylobacter hominis*; B: *Bacillus cereus*; C: *Dyadobacter koreensis*; D: *Pseudomonas aeruginosa*; E: *Micrococcus luteus*

Amplification of specific primer for *nahH* gene by hydrocarbon-degrading bacteria is shown in Figure 8. The primer was amplified by *Bacillus cereus, Dyadobacter koreensis, Pseudomonas aeruginosa* and *Micrococcus luteus.* The positive bands of 900 bp were considered to demonstrate the existence of *nahH* gene. However, there was no amplification with the *CatA* gene primer by the isolates thus showing the absence of the *CatA* gene in all the isolates (Figure 9). As shown in Figure 10, only three isolates: *Pseudomonas aeruginosa, Bacillus cereus* and *Dyadobacter koreensis* DNA were amplified to give 260 bp amplicons indicating the presence of the *alkB* gene while *Pseudomonas aeruginosa* and *Micrococcus luteus* DNA were not amplified.

## 4. Discussion

Petroleum hydrocarbons remain the most important energy and chemical source as well as the most challenging organic pollutants in future [24]. Microbial strains have been isolated from specific contaminated environments have shown bioremediation potentials and as such offers a green alternative approach to clean up these hazardous environmental pollutants [12],[25],[26]. Five hydrocarbon degrading species isolated from Agbabu bitumen deposit in Ondo State were employed in this study to degrade PAHs present in crude oil. Isolates from bitumen sites have been known to possess crude oil degradation abilities [17].

The gas chromatography-mass spectrometry (GS-MS) analysis of degraded crude oil revealed the presence of the 16 US Environmental Protection Agency (EPA) PAHs at varying proportions. The low and medium molecular weight PAHs are the major components of the crude oil while the high molecular weight PAHs form the minor part. Many of these PAHs are known to be toxic and carcinogenic to humans and their contamination of soils and aquifers is of great environmental concern [27]. However, treatment of crude oil with the selected hydrocarbon-degrading bacteria resulted in drastic reduction of these PAH components of the crude oil. This indicates the stored potential to degrade PAH contaminants and presence of functional genes is prevalent in bacteria indigenous to Agbabu bitumen deposit. Meanwhile, there was variation in the rate of degradation of PAH components of the crude oil by the isolates. It has been reported that microorganisms are known to degrade specific components of the crude oil [28]–[30]. It has also been observed that different compounds in the crude oil samples were degraded to a different extent by the same organisms, indicating that the bioavailability of a particular compound in a crude oil sample may be the determining factor for effective biodegradation of the compound [29].

Treatment of crude oil with *P*. *aeruginosa* resulted in reduction of all the PAH components of the crude oil. Significant depletion of the high-molecular weight PAHs fractions, (Chrysene, Benzo (α) pyrene indeno (1, 2, 3-cd) pyrene benzo (g, h, i) perylene) of the crude oil was also observed. Similarly, significant reduction in the concentrations of all the PAH fractions, especially the heavy PAHs was observed in *Dyadobacter koreensis* treated sample. These high molecular weight compounds, are major contaminant classes of concern in oil spills because they are known for their mutagenic, teratogenic properties and are toxic and/or carcinogenic to humans and wildlife and are often recalcitrant to degradation in environment for example, benzo (α) pyrene metabolites are mutagenic and highly carcinogenic and it is listed as a Group 1 carcinogen by the IARC (The International Agency for Research on Cancer [31].

The decrease in the proportion of heavy PAHs and a concomitant increase in the proportion of lighter ones observed in the study could be as a result of heavier fractions in the oil reducing to lighter fractions, thereby increasing the concentration of lighter PAH fractions. Similar findings were reported by other investigators with a decrease in the concentrations of heavy PAHs which resulted to an increase in the concentrations of lighter PAH compounds in residual crude oil by *Pseudomonas* sp., *Actinomyces* sp. and *Bacillus* sp [32]–[34].

Treatment of the crude oil with consortium resulted in higher degradation of the PAH components as compared to individual strains. Inoculation of the combination of the five hydrocarbon-degrading bacteria (Consortium D) resulted in the total removal of Indeno (1, 2, 3, c d) pyrene, Benzo (k) fluoranthene, Benzo (b) fluoranthene and dibenzo (a, h) anthracene and significant reduction in the concentrations of other PAHs. This result indicated that the addition of the mixed bacteria culture enhanced the rate of degradation of the aromatic fractions of the crude oil. Kumari *et al*. [35] also confirmed in their investigation that consortium proves a better model for degradation than single cultures. Several reports have also reported the enhanced degradation of mixed PAHs, by a bacterial consortium because of their enzymatic and metabolic function was stimulated by the communities involved as consortium rather than single strain [36]–[39].

In a study conducted by Ghorbannezhad *et al.* [40], the total petroleum hydrocarbons degradation rates of strains S1, S2, and S3 were 14.28%,10.68%, and 15.67%, respectively; however, the synergistic effect of the three bacterial strains produced higher total petroleum hydrocarbon degradation (19.59%), which significantly improved crude oil biodegradation, when compared with the use of single strains. Effective degradation of pollutants was achieved through symbiosis and synergy among different strains, generating complex microbial flora and significantly improving the available range and utilization efficiency of petroleum-based matrix for the purpose of degradation [41].

Vinithini *et al.* [42] also emphasized that there is no single strain of bacteria with the metabolic capacity to degrade all the components found within crude oil. Also, in nature, biodegradation of crude oil typically involves a succession of species within the consortia of microbes present. Thus, this present investigation corroborates the potentials of microbial consortium for biodegradation of PAHs present in crude oil exploiting them for the remediation of PAHs contaminated sites.

The PCR amplification of specific primers for *nahH* gene in these organisms revealed that *nahH* gene was present in *B. cereus, D. koreensis, P. aeruginosa* and *M. luteus*. This agrees with several other reports which showed the detection of this catabolic gene (*nahH*) in numerous Gram-negative (*Pseudomonas* spp., *Sphingomonas* spp., *Acinetobacter* spp., *Ralstonia* spp., *Burkholderia* spp. and Gram-positive (*Nocardia* spp., *Rhodococcus* spp. and *Bacillus* spp.) bacteria [43],[44]. Several researchers have assessed the degradation of hydrocarbons by environmental microflora and reported that it involves having specialized metabolic capabilities and the presence of hydrocarbon degradative genes like aromatic ring cleavage dioxygenases (*nahH* and *CatA*) genes. The ability of an organism to degrade a specific substrate is clear evidence that its genome harbors the relevant degrading gene. The result from this study gives credence to the fact that these bacterial genera which showed positive bands of 900 bp for detection of *nahH* gene are well adapted to the polluted environment. The detection of the relevant degradative gene (*nahH*) in the indigenous bacteria in this hydrocarbon-impacted area shows that they have the natural propensity to degrade hydrocarbons. The *nahH* gene codes for catechol 2, 3-dioxygenase which is an enzyme that catalyses the breakdown of aromatic hydrocarbons through a meta-cleavage pathway to produce metabolites which enter the TCA cycle [45].

However, none of the isolates amplified primer specific for *CatA* gene. This implies the absence of this gene in all the organisms. The *CatA* gene codes for catechol 1, 2-dioxygenase an enzyme responsible for the mineralization of aromatic compounds via *ortho*-cleavage pathway. Since the organisms harbored only gene (*nahH)* coding for catechol 2, 3-dioxygenase, it could be inferred that the degradation of aromatic fractions of crude oil by these organisms was via the *meta*-cleavage pathway. It has been reported that the o*rtho* pathway catalyze completed degradation of hydrocarbons while the *meta* pathway is known for incomplete metabolism due to production of dead-end or suicide metabolites [46]. This probably accounts for the incomplete mineralization of the poly aromatic components of the crude oil in this study.

The alkane monooxygenase, which is encoded by the *alkB* gene, is a key enzyme involved in bacterial alkane degradation. The *alkB* gene was present in only three (*Pseudomonas aeruginosa, Bacillus cereus* and *Dyadobacter koreensis*) while it was not detected in the other two (*Campylobacter hominis* and *Micrococcus luteus*) organisms (Figure 10). The ability of the selected hydrocarbon-degrading bacteria to degrade aliphatic fractions of the crude oil could be attributed to the presence of this gene.

Catabolic pathways, which encode degradation routes of different aromatic and aliphatic hydrocarbons are frequently located on plasmids, although degradative genes can be located on either chromosome or plasmid [47]. Since these dioxygenase genes are commonly distributed between plasmids of different sizes [48], it could be presumed that these genes may spread to different bacteria through horizontal gene transfer.

## 5. Conclusion

Five bacterial isolates employed in this study were able to biodegrade the PAHs component of Bonny Light crude oil with the consortium combinations being found to be a more efficient system to achieve synergistic enhanced rates for PAHs degradation. Thus, the isolated strains possess potential applications in the remediation of crude oil contaminated soil as well treatment of bitumen spills which was evident in Agbabu community in Ondo State where large deposit of bitumen is found in Nigeria.

## References

[1] Oboh BO, Ilori OM, Akinyemi JO (2006). Hydrocarbon degrading potentials of bacteria isolated from a Nigeria Bitumen (Tarsand) deposit. Nat Sci.

[2] Kadafa AA (2012). Environmental impacts of oil exploration and exploitation in the Niger Delta of Nigeria. Global J Sci Front Res Environ Earth Sci.

[3] F M E (2006). Niger Delta natural resource damage assessment and restoration project: Phase 1-Scoping report. Res J Environ Toxicol.

[4] Bidoia ED, Montagnolli RN, Lopes PRM, Mendez-Vilas A (2010). Microbial biodegradation potential of hydrocarbons evaluated by colorimetric technique: a case study. Current research, technology and education topics in applied microbiology and microbial biotechnology.

[5] Mwaura AN, Mbatia BN, Muge EK (2018). Screening and characterization of hydrocarbonoclastic bacteria isolated from oil-contaminated soils from auto garages. Int J Microbiol Biotechn.

[6] Boonchan S, Britz ML, Stanley GA (2000). Degradation and mineralization of high-molecular-weight polycyclic aromatic hydrocarbons by defined fungal-bacterial co-cultures. Appl Environ Microbiol.

[7] Mao J, Luo Y, Teng Y (2012). Bioremediation of polycyclic aromatic hydrocarbon-contaminated soil by a bacterial consortium and associated microbial community changes. Int Biodeterior Biodegrad.

[8] Lam SS, Russell AD, Lee CL (2012). Production of hydrogen and light hydrocarbons as a potential gaseous fuel from microwave-heated pyrolysis of waste automotive engine oil. Int J Hydrogen Energ.

[9] Islas-garcia A, Vega-loyo L, Aguilar-lopez R (2015). Evaluation of hydrocarbons and organochlorine pesticides and their tolerant microorganisms from an agricultural soil to define its bioremediation feasibility. J Environ Sci Health B.

[10] Watanabe K (2001). Microorganisms relevant to bioremediation. Curr Opin Biotechnol.

[11] Bushnell LD, Haas HF (1941). The utilization of hydrocarbons by microorganisms. J Bacteriol.

[12] Oluwole OA, Oluyege JO, Odeyemi AT (2017). Growth patterns and degradative potentials of *Pseudomonas* sp. isolated from waste dumpsite soil in crude oil supplemented soil extract and mineral salts media. J Adv Biolo Biotechnol.

[13] Margesin R, Schinner F (2001). Bioremediation (natural attenuation and biostimulation) of diesel-oil-contaminated soil in Alpine glacier sking area. Appl Environ Microbiol.

[14] Semple KT, Morriss A, Paton GI (2003). Bioavailability of hydrophobic organic contaminants in soils: fundamental concepts and techniques for analysis. Eur J Soil Sci.

[15] Sathishkumar M, Arthur RB, Sang-Ho B (2008). Biodegradation of crude oil by individual bacterial strains and a mixed bacterial consortium isolated from hydrocarbon contaminated areas. Clean.

[16] Obayori OS, Ilori MO, Adebusoye SA (2009b). Degradation of hydrocarbons and biosurfactant production by *Pseudomonas* sp. strain LP1. World J Microb Biot.

[17] Olabemiwo MO, Adediran GO, Adekola FA (2014). Biodegradation of hydrocarbon compounds in Agbabu natural bitumen. Afr J Biotechnol.

[18] Olowomofe Temitayo O, Oluyege JO, Olawole OA (2018). Catechol-2,3-dioxygenase and Lipase Activities during Degradation of Crude Oil by Hydrocarbon-degrading Bacteria Isolated from Bitumen-polluted Surface Water in Agbabu, Ondo State. Int J Environ Biorem Biodegrad.

[19] Olowomofe TO, Oluyege JO, Sowole DO (2017). Isolation, screening and characterization of hydrocarbon-utilizing bacteria isolated from bitumen-contaminated surface water in Agbabu, Ondo State. J Adv Biol Biotechnol.

[20] Orhorhoro E, Effiong E, Abu G (2018). Laboratory-scale bioremediation of crude oil polluted soil using a consortia of rhizobacteria obtained from plants in Gokana-Ogoni, Rivers State. J Adv Microbiol.

[21] Minai-Tehrani D, Herfatmanesh A (2007). Biodegradation of aliphatic and aromatic fractions of heavy crude oil-contaminated soil: a pilot study. J Biodegrad Biorem.

[22] Baek KH, Yoon BD, Oh HM (2006). Biodegradation of aliphatic and aromatic hydrocarbons by Nocardia sp. H17–1. Geomicrobiology J.

[23] Tancsics A, Szabó I, Baka E (2008). Investigation of catechol 2,3-dioxygenase and 16S rRNA gene diversity in hypoxic, petroleum hydrocarbon contaminated groundwater. Syst Appl Microbiol.

[24] Khan MA, Biswas B, Smith E (2018). Toxicity assessment of fresh and weathered petroleum hydrocarbons in contaminated soil. Chemosphere.

[25] Aurepatipan N, Champreda V, Kanokratana P (2018). Assessment of bacterial communities and activities of thermotolerant enzymes produced by bacteria indigenous to oil-bearing sandstone cores for potential application in Enhanced Oil Recovery. J Petroleum Sci.

[26] Ji H, Gong Y, Duan J (2018). Degradation of petroleum hydrocarbons in seawater by simulated surface level atmospheric ozone: reaction kinetics and effect of oil dispersant. Mar Pollut Bull.

[27] Gupta G, Kumar V, Pal AK (2016). Biodegradation of polycyclic aromatic hydrocarbons by microbial consortium: a distinctive approach for decontamination of soil. Soil Sediment Contam.

[28] Das K, Mukherjee F (2007). Crude petroleum-oil biodegradation efficiency of *Bacillus subtilis* and *Pseudomonas aeruginosa* strains isolated from a petroleum-oil contaminated soil from North-East India. Bioresour Technol.

[29] Amini S, Zulkify AH (2011). Molecular identification and characterization of a bacterium that has potential to degrade low concentration of halo alkanoic acid. Res J Microbiol.

[30] Olabemiwo OM, Adediran GO, Adekola FA (2011). Impact of simulated Agbabu bitumen Leachate on haematological and biochemical parameters of wistar albino rat. Res J Environ Toxicol.

[31] International Agency for Research on Cancer (IARC) (2010). Some non-heterocyclic polycyclic aromatic hydrocarbons and some related exposures. Int Agency Res Cancer.

[32] Deng D, Li C, Ju Q (1999). Systematic extensive laboratory studies of microbial EOR mechanisms and microbial EOR application results in Changing Oilfield. SPE Asia Pacific Oil and Gas Conference and Exhibition; Society of Petroleum Engineers.

[33] Etoumi A, Musrati IE, Gammoudi BE (2008). The reduction of wax precipitation in waxy crude oils by *Pseudomonas* species. J Ind Microbiol Biotechnol.

[34] Al-Sayegh A, Al-Wahaibi Y, Al-Bahry S (2015). Microbial enhanced heavy crude oil recovery through biodegradation using bacterial isolates from an Omani oil field. Microb Cell Fact.

[35] Kumari S, Regar RK, Manickam N (2018). Improved polycyclic aromatic hydrocarbon degradation in a crude oil by individual and a consortium of bacteria. Bioresour Technol.

[36] Anastasi A, Coppola T, Prigione V (2009). Pyrene degradation and detoxification in soil by a consortium of basidiomycetes isolated from compost: Role of laccases and peroxidases. J Hazard Mater.

[37] Wu M, Chen L, Tian Y (2013). Degradation of polycyclic aromatic hydrocarbons by microbial consortia enriched from three soils using two different culture media. Environ Pollut.

[38] Zafra G, Absalón AE, Cortés-Espinosa DV (2015). Morphological changes and growth of filamentous fungi in the presence of high concentrations of PAHs. Braz J Microbiol.

[39] Wanapaisan P, Laothamteep N, Vejarano F (2018). Synergistic degradation of pyrene by five culturable bacteria in a mangrove sediment-derived bacterial consortium. J Hazard Mater.

[40] Ghorbannezhad H, Moghimi H, Taheri RA (2018). Enhanced biodegradation of phenol by magnetically immobilized *Trichosporon cutaneum*. Ann Microbiol.

[41] Owsianiak M., Szulc A, Chrzanowski L (2009). Biodegradation and surfactant-mediated biodegradation of diesel fuel by 218 microbial consortia are not correlated to cell surface hydrophobicity. Appl Microbiol Biot.

[42] Vinithini C, Sudhakar S, Ravikumar R (2015). Biodegradation of petroleum and crude oil by *Pseudomonas putida* and *Bacillus cereus*. Int J Curr Microbiol Appl Sci.

[43] Ma Y, Wang L, Shao Z (2006). Pseudomonas, the dominant polycyclic aromatic hydrocarbon-degrading bacteria isolated from Antarctic soils and the role of large plasmids in horizontal gene transfer. Natl Cent Biotechnol Inf.

[44] Jyothi KK, Surendra B, Nancy CK (2012). Identification and Isolation of Hydrocarbon-degrading Bacteria by Molecular Characterization. Helix.

[45] El-Sayed WS (2006). Molecular cloning of gene nahH encoding extradiol-type dioxygenase from the NAH plasmid of *Pseudomonas stutzeri* NA1. Ann Microbiol.

[46] Veenagayathri K, Vasudevan N (2011). Ortho and meta cleavage dioxygenases detected during the degradation of phenolic compounds by a moderately halophilic bacterial consortium. Int Res J Microbiol.

[47] Zhao K, Guo X, Gong JA (2013). Novel benzoate-degrading Rhodococcus strain contains three catA genes with one being transcriptionally active during the growth on benzoate. Res J Environ Earth Sci.

[48] Dokic L, Narancic T, Nikodinovic-Runic J (2011). Four Bacillus sp. soil isolates capable of degrading phenol, toluene, biphenyl, naphthalene and other aromatic compounds exhibit different aromatic catabolic potentials. Arch Biolo Sci.

